# Angular Rate Sensing with GyroWheel Using Genetic Algorithm Optimized Neural Networks

**DOI:** 10.3390/s17071692

**Published:** 2017-07-22

**Authors:** Yuyu Zhao, Hui Zhao, Xin Huo, Yu Yao

**Affiliations:** Control and Simulation Center, Harbin Institute of Technology, Harbin 150080, China; zhaohui@hit.edu.cn (H.Z.); huoxin@hit.edu.cn (X.H.); yaoyu@hit.edu.cn (Y.Y.)

**Keywords:** GyroWheel, angular rate sensing, large tilt angles, genetic algorithm, artificial neural network

## Abstract

GyroWheel is an integrated device that can provide three-axis control torques and two-axis angular rate sensing for small spacecrafts. Large tilt angle of its rotor and de-tuned spin rate lead to a complex and non-linear dynamics as well as difficulties in measuring angular rates. In this paper, the problem of angular rate sensing with the GyroWheel is investigated. Firstly, a simplified rate sensing equation is introduced, and the error characteristics of the method are analyzed. According to the analysis results, a rate sensing principle based on torque balance theory is developed, and a practical way to estimate the angular rates within the whole operating range of GyroWheel is provided by using explicit genetic algorithm optimized neural networks. The angular rates can be determined by the measurable values of the GyroWheel (including tilt angles, spin rate and torque coil currents), the weights and the biases of the neural networks. Finally, the simulation results are presented to illustrate the effectiveness of the proposed angular rate sensing method with GyroWheel.

## 1. Introduction

The development of small spacecraft has received a lot of attention in recent years [1,2,3,4]. Small spacecraft designers face difficult hurdles such as mass, power and volume constraints, which significantly impact its cost. It inherently makes sense that some subsystems could be combined where possible into a lighter, cheaper, smaller, commercially available and proven system [5,6,7]. GyroWheel, a novel attitude determination and control system [7], is such a multi-function system, and it offers the potential to meet the constraints and cost requirements for small spacecrafts. It provides control torques about three axes, and measures the spacecraft angular rates about the two axes perpendicular to the spin directions simultaneously [8,9], which improves the integration and efficiency of attitude control system in small spacecrafts.

The conception of GyroWheel is inspired by a dynamically tuned gyroscope (DTG). Compared with a DTG, it has a larger rotor and tilt angles, as well as a time-varying spin rate due to its multi-function capability. For a DTG, the tilt of the rotor with respect to its case is regulated about null, and it is operated in the tuned condition all the time [10,11,12]. Therefore, the control torque required to maintain its zero tilt is a direct measure of the external angular rates of the DTG’s case about the two orthogonal axes lying in the rotor radial plane. The rate sensing principle of the DTG is quite simple. However, GyroWheel is frequently operated in a de-tuned condition and at a non-zero tilt. The control torque required to hold the rotor spin axis at a constant tilt angle is a function of two independent effects: the torque induced by the spacecraft angular rates and the torque required to overcome the de-tuned stiffness, damping and gyroscopic coupling [8]. Hence, it is more complicated to measure spacecraft angular rates with GyroWheel.

A few studies about angular rate sensing with GyroWheel have been conducted. Ower at Carleton University derived a three-body, non-linear dynamic model of the GyroWheel based on Lagrange’s equations, and developed a rate sensing method by linearizing the non-linear model around the operating point of zero spin axis tilt with a constant spin rate [8]. However, the measurement error rises significantly with the increase of the tilt angles due to the linearization at zero tilt, which limits its application in the large tilt conditions. Considering the rate sensing problems under large tilt conditions, the linearization errors were compensated with polynomial functions of tilt angles, and a real-time linearization model at arbitrary tilt angles was proposed [13]. Both approaches requires an exact knowledge of the GyroWheel parameters, such as the moments of inertia, the torque factors, the stiffness and the damping coefficients, the majority of which are difficult to identify. Hence the error compensation and real-time linearization are limited in practical engineering. In order to improve the measurement accuracy of the GyroWheel, Hall studied the problems of GyroWheel calibration, which was used to avoid the influence of changes in temperature and gravitational acceleration [14]. In addition, a D-optimal multi-position calibration method was developed to reduce the influence of random noises [15,16]. However, neither the accuracy loss caused by zero tilt linearization nor the identification of system parameters has been considered in these studies.

Actually, the challenges to realize rate sensing with GyroWheel consist in the complexity and its non-linear model, and the identification of the model parameters. artificial neural networks (ANN) have been successfully applied to solve problems involving modeling complex dynamic systems [17,18,19,20]. ANNs are parallel computing systems inspired by biological neural networks. They can be defined as a set of elementary processing units that communicate with each other by weighted connections. Each unit receives input signals from near units or external sources and gives an output signal, which propagates to other units or constitutes a part of the network output [21]. The ANN-based models differ from conventional models, as they are based on experimental data rather than theoretical derivations [22,23]. Therefore, a soft sensor using an ANN as a black box model can be used to obtain an estimate of a variable in complex dynamic systems [20]. Besides, global optimization algorithms are often added to the training process of the ANNs to avoid local minima, which leads to false convergence of the ANN models [22,24,25,26]. Motivated by these facts, the problem of angular rate sensing with GyroWheel is investigated in this paper. A practical rate sensing approach is presented based on genetic algorithm (GA) optimized neural networks, which can be applied to measure angular rates within the whole operating range of GyroWheel.

The remainder of this paper is as follows. In Section 2, the mechanical configuration of the GyroWheel system is described, and the dynamic equations are derived by applying the Lagrange equations of the second kind. In Section 3, a linearization of the dynamic model is performed for the purpose of rate sensing, and the characteristics of the rate sensing errors are analyzed. In Section 4, according to the error analysis results, a rate sensing principle based on torque balance theory is developed. Using explicit genetic algorithm optimized neural networks, a practical way to estimate the angular rates with the GyroWheel is provided. Then simulations are performed to illustrate the effectiveness of the proposed rate sensing method. Section 5 concludes this paper and outlines areas for future studies.

## 2. Overview of GyroWheel System

### 2.1. GyroWheel Mechanical Configuration

**Figure 1:**
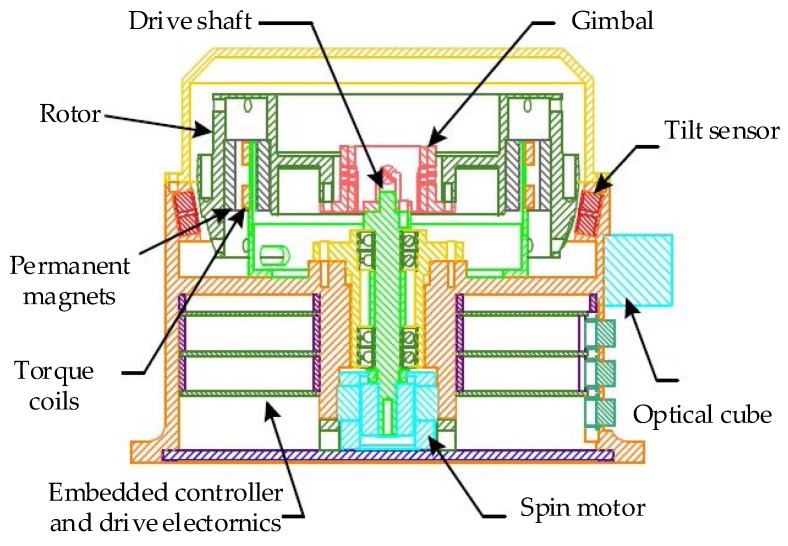
Cross-sectional view of the GyroWheel system.

GyroWheel is a novel attitude determination and control system. The structure of GyroWheel is inspired by a DTG, but it has a significantly larger rotor compared to the classical DTG. It is a mechanism based on the fundamental law of precession, which is the most common method of measuring angular rotations. To provide control torques, its spinning rate is constantly varying and it is always operated at large tilt angles. In this sense, it is a form of double gimbaled control moment gyroscope (CMG), but based on using a spinning flex-gimbal system as opposed to the usual non-spinning motor driven gimbals that are typically used in CMG torque actuators. A cross-sectional view of the GyroWheel system is shown in Figure 1 [8,14]. The system consists of the following components: case, spin motor, gimbal assembly, rotor, tilt sensors, permanent magnets, torque coils, controller and drive electronics.

The gimbal assembly consists of a hollow cylindrical section with two pairs of crossed-flexure pivots. The rotor is coupled to the drive shaft of the spin motor through the gimbal assembly. A brushless direct current (DC) motor spins the gimbal and the rotor, and the motor is designed as part of the GyroWheel case. Torque coils that are fixed to the stationary case, are used to interact with permanent magnets mounted in the rotor to allow steering the rotor angular momentum vector. The tilt angles of the rotor is measured by non-contact sensors located at 90 degree intervals around the case. In order to measure the external angular rates, the GyroWheel system is used in a torque rebalance mode. The tilt sensor signals are amplified and delivered to the rebalance loop which processes the signal and produces a torque command which is delivered to the torque coil. This causes the torque coil to apply a torque to the rotor so as to maintain the rotor in the desired position.

### 2.2. Dynamic Models of GyroWheel System

**Figure 2:**
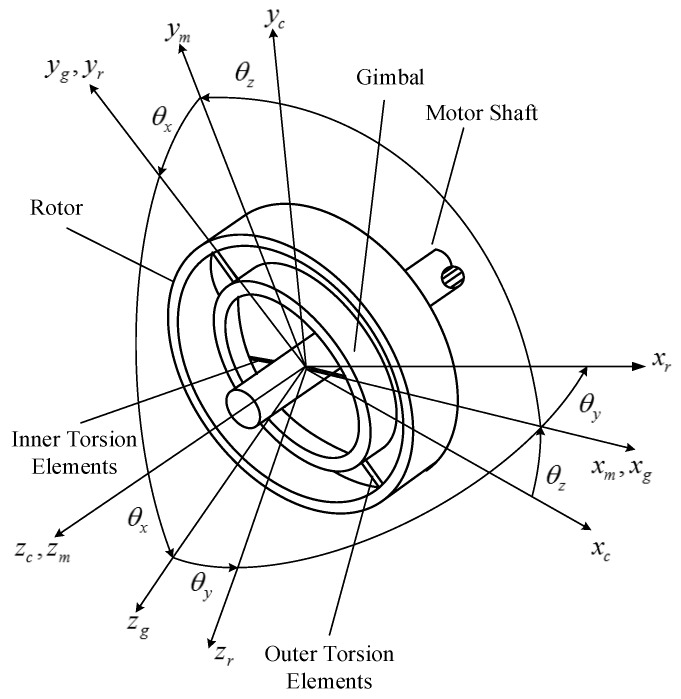
Reference frames and gimbal angles.

As seen in Figure 1, the GyroWheel system consists of three bodies: a motor shaft, a gimbal and a rotor. Four body-fixed reference frames are defined to facilitate the derivation of dynamic models. They are the case frame ($F_{c}:O-x_{c}y_{c}z_{c}$), the motor frame ($F_{m}:O-x_{m}y_{m}z_{m}$), the gimbal frame ($F_{g}:O-x_{g}y_{g}z_{g}$), and the rotor frame ($F_{r}:O-x_{r}y_{r}z_{r}$). The case frame $F_{c}$ is attached to the spacecraft body. Motion of the three bodies is constrained except about three gimbal angles $\theta_{x},\;\theta_{y}$ and $\theta_{z}$, as shown in Figure 2.

According to Figure 2, the attitude of the rotor can be described by the following gimbal-referenced rotation sequence: a rotation $\theta_{z}$ about the $z_{c}$-axis, followed by a rotation $\theta_{x}$ about the $x_{m}$-axis and then a rotation $\theta_{y}$ about the $y_{g}$-axis.

The spacecraft angular velocity respect to the inertial frame is expressed in the case frame $F_{c}$:

(1) $$\omega_{c}={\left[\begin{array}{ccc} \omega_{cx} & \omega_{cy} & \omega_{cz} \end{array}\right]}^{T}$$

According to the above rotation sequence, the angular velocity of the motor shaft expressed in the motor frame $F_{m}$ is given by:

(2) $$\omega_{m}={\left[\begin{array}{ccc} \omega_{mx} & \omega_{my} & \omega_{mz} \end{array}\right]}^{T}={\left[\begin{array}{ccc} 0 & 0 & {\dot{\theta }}_{z} \end{array}\right]}^{T}+T_{\theta_{z}}\cdot \omega_{c}$$

Similarly, the gimbal angular velocity expressed in the gimbal frame $F_{g}$, and the rotor angular velocity expressed in the rotor frame $F_{r}$ can be calculated as following:

(3) $$\omega_{g}={\left[\begin{array}{ccc} \omega_{gx} & \omega_{gy} & \omega_{gz} \end{array}\right]}^{T}={\left[\begin{array}{ccc} {\dot{\theta }}_{x} & 0 & 0 \end{array}\right]}^{T}+T_{\theta_{x}}\cdot \omega_{m}$$

(4) $$\omega_{r}={\left[\begin{array}{ccc} \omega_{rx} & \omega_{ry} & \omega_{rz} \end{array}\right]}^{T}={\left[\begin{array}{ccc} 0 & {\dot{\theta }}_{y} & 0 \end{array}\right]}^{T}+T_{\theta_{y}}\cdot \omega_{g}$$

where $T_{\theta_{x}},\;T_{\theta_{y}},\;T_{\theta_{z}}$ are rotation matrixes, which describe the transform relations between adjacent frames.

The Lagrangian for the GyroWheel system can be defined by:

(5) L=T−V

where *T* is the total kinetic energy of the system, and *V* is the potential energy of the system. They take the following form:

(6) $$\begin{array}{l} T=\frac{1}{2}\left(\sum_{i=x,y,z}I_{mi}\omega_{mi}^{2}+\sum_{i=x,y,z}I_{gi}\omega_{gi}^{2}+\sum_{i=x,y,z}I_{ri}\omega_{ri}^{2}\right)\\ V=\frac{1}{2}\left(K_{x}\theta_{x}^{2}+K_{y}\theta_{y}^{2}\right) \end{array}$$

where $K_{x},\;K_{y}$ are the stiffness coefficients of the two crossed-flexure pivot pairs. $I_{mi},\;I_{gi},\;I_{ri},\;i=x,y,z$ are the moments of inertia of the motor shaft, the gimbal and the rotor respectively. To simplify the derivation without loss of accuracy, the transverse inertias and spin inertias are rewritten as:

(7) $$I_{gx}=I_{gy}=I_{gt},\;I_{rx}=I_{ry}=I_{rt},\;I_{gz}=I_{gs},\;I_{rz}=I_{rs}$$

To derive the dynamic models of the GyroWheel system, $\left(\theta_{x},\theta_{y},\theta_{z}\right)$ are defined as the generalized coordinates. Then the dynamic models can be determined by applying the Lagrange equations of the second kind:

(8) $$\begin{array}{l} \frac{d}{dt}\left(\frac{\partial L}{\partial {\dot{\theta }}_{x}}\right)-\frac{\partial L}{\partial \theta_{x}}=T_{gx}-C_{x}{\dot{\theta }}_{x}\\ \frac{d}{dt}\left(\frac{\partial L}{\partial {\dot{\theta }}_{y}}\right)-\frac{\partial L}{\partial \theta_{y}}=T_{gy}-C_{y}{\dot{\theta }}_{y}\\ \frac{d}{dt}\left(\frac{\partial L}{\partial {\dot{\theta }}_{z}}\right)-\frac{\partial L}{\partial \theta_{z}}=T_{gz} \end{array}$$

where $T_{gx},\;T_{gy},\;T_{gz}$ are the generalized control torques acting about the three gimbal axes. $C_{x},\;C_{y}$ are the damping coefficients of the two crossed-flexure pivot pairs, and $C_{x}=C_{y}=C_{g}$.

By calculating the partial derivative terms in Equation (8), the dynamic models can be expressed in the following form:

(9) $$M\left(x\right)\ddot{x}+C\dot{x}+Kx=T_{g}+F_{nl}\left(x,\dot{x},\omega_{c},{\dot{\omega }}_{c}\right)$$

where

$$\begin{array}{l} x={\left[\begin{array}{ccc} \theta_{x} & \theta_{y} & \theta_{z} \end{array}\right]}^{T},\;T_{g}={\left[\begin{array}{ccc} T_{gx} & T_{gy} & T_{gz} \end{array}\right]}^{T}\\ M\left(x\right)=\left[\begin{array}{ccc} I_{1} & 0 & I_{2}\\ 0 & I_{rt} & I_{rt}S_{\theta_{x}}\\ I_{2} & I_{rt}S_{\theta_{x}} & I_{3} \end{array}\right],\;C=\left[\begin{array}{ccc} C_{g} & 0 & 0\\ 0 & C_{g} & 0\\ 0 & 0 & 0 \end{array}\right],\;K=\left[\begin{array}{ccc} K_{x} & 0 & 0\\ 0 & K_{y} & 0\\ 0 & 0 & 0 \end{array}\right]\\ F_{nl}\left(x,\dot{x},\omega_{c},{\dot{\omega }}_{c}\right)={\left[\begin{array}{ccc} f_{nlx}\left(x,\dot{x},\omega_{c},{\dot{\omega }}_{c}\right) & f_{nly}\left(x,\dot{x},\omega_{c},{\dot{\omega }}_{c}\right) & f_{nlz}\left(x,\dot{x},\omega_{c},{\dot{\omega }}_{c}\right) \end{array}\right]}^{T}\\ I_{1}=I_{rt}C_{\theta_{y}}^{2}+I_{rs}S_{\theta_{y}}^{2}+I_{gt}\\ I_{2}=\frac{1}{2}\left(I_{rs}-I_{rt}\right)C_{\theta_{x}}S_{2\theta_{y}}\\ I_{3}=I_{rt}C_{\theta_{x}}^{2}S_{\theta_{y}}^{2}+I_{rt}S_{\theta_{x}}^{2}+I_{rs}C_{\theta_{x}}^{2}C_{\theta_{y}}^{2}+I_{gt}S_{\theta_{x}}^{2}+I_{gs}C_{\theta_{x}}^{2}+I_{mz} \end{array}$$

The detailed expressions for the non-linear torque functions can be found in Reference [8].

## 3. GyroWheel Rate Sensing for Small Tilt Conditions and Error Analysis

### 3.1. Rate Sensing Equation for Small Tilt Conditions

A linearization of the dynamic Equation (9) is required for the purpose of rate sensing. The linearization occurs about the nominal operating condition of zero tilt with a constant spin rate $\omega_{s}$, where

(10) $$x_{0}=\left[\begin{array}{c} 0\\ 0\\ \omega_{s}t \end{array}\right],\;{\dot{x}}_{0}=\left[\begin{array}{c} 0\\ 0\\ \omega_{s} \end{array}\right],\;{\ddot{x}}_{0}=\left[\begin{array}{c} 0\\ 0\\ 0 \end{array}\right]$$

According to Lyapunov’s linearization method, the dynamic equation can be expressed in the following form:

(11) $$\begin{array}{l} \;\frac{\partial \left(M\ddot{x}\right)}{\partial \ddot{x}}\left(\ddot{x}-{\ddot{x}}_{0}\right)+\frac{\partial \left(C\dot{x}\right)}{\partial \dot{x}}\left(\dot{x}-{\dot{x}}_{0}\right)+\frac{\partial \left(Kx\right)}{\partial x}\left(x-x_{0}\right)\\ =T_{g}+{\frac{\partial F_{nl}}{\partial x}|}_{\begin{array}{l} x=x_{0},\dot{x}={\dot{x}}_{0},\\ \omega_{c}=0,\omega_{c}=0 \end{array}}\left(x-x_{0}\right)+{\frac{\partial F_{nl}}{\partial \dot{x}}|}_{\begin{array}{l} x=x_{0},\dot{x}={\dot{x}}_{0},\\ \omega_{c}=0,\omega_{c}=0 \end{array}}\left(\dot{x}-{\dot{x}}_{0}\right)+{\frac{\partial F_{nl}}{\partial \omega_{c}}|}_{\begin{array}{l} x=x_{0},\dot{x}={\dot{x}}_{0},\\ \omega_{c}=0,\omega_{c}=0 \end{array}}\omega_{c}+{\frac{\partial F_{nl}}{\partial {\dot{\omega }}_{c}}|}_{\begin{array}{l} x=x_{0},\dot{x}={\dot{x}}_{0},\\ \omega_{c}=0,\omega_{c}=0 \end{array}}{\dot{\omega }}_{c}+F_{h.o.t}\left(x,\dot{x},\omega_{c},{\dot{\omega }}_{c}\right) \end{array}$$

Ignoring the higher order terms $F_{h.o.t}\left(x,\dot{x},\omega_{c},{\dot{\omega }}_{c}\right)$, Equation (9) is simplified to a linearized dynamic equation.

Note that the control torques are generated and the rotor tilt angles are measured with respect to the case. Therefore, it is necessary to describe the motion of the rotor with respect to the case frame. The relationship between the case-referenced angles $\left(\varphi_{x},\varphi_{y}\right)$ and the gimbal angles $\left(\theta_{x},\theta_{y}\right)$ is given by:

(12) $$\left[\begin{array}{c} \varphi_{x}\\ \varphi_{y} \end{array}\right]=\left[\begin{array}{cc} \cos\left(\omega_{s}t\right) & -\sin\left(\omega_{s}t\right)\\ \sin\left(\omega_{s}t\right) & \cos\left(\omega_{s}t\right) \end{array}\right]\left[\begin{array}{c} \theta_{x}\\ \theta_{y} \end{array}\right]$$

Without loss of accuracy, $2\omega_{s}$ cyclic terms induced by frame transformation and angular acceleration terms are ignored, the rate sensing equation of the GyroWheel system can be written as:

(13) $$\begin{array}{l} \omega_{cx}=\frac{1}{h_{s}}\left(-T_{cy}+C_{g}{\dot{\varphi }}_{y}+K_{d}\varphi_{y}-I_{d}\omega_{s}{\dot{\varphi }}_{x}-C_{g}\omega_{s}\varphi_{x}\right)\\ \omega_{cy}=\frac{1}{h_{s}}\left(T_{cx}-C_{g}{\dot{\varphi }}_{x}-K_{d}\varphi_{x}-I_{d}\omega_{s}{\dot{\varphi }}_{y}-C_{g}\omega_{s}\varphi_{y}\right) \end{array}$$

where $h_{s}=\left(I_{rs}+\frac{I_{gs}}{2}\right)\omega_{s},\;K_{d}=\frac{K_{x}+K_{y}}{2}-\left(I_{gt}-\frac{1}{2}I_{gs}\right)\omega_{s}^{2},\;I_{d}=I_{rs}+I_{gt}$, $T_{cx},\;T_{cy}$ are control torques described in the case frame.

To ensure rate sensing accuracy, the GyroWheel is applied as a time-sharing system [13]. It has two working modes: actuator mode and sensor mode. When the GyroWheel is used as a sensor to measure spacecraft angular rates, the tilt angles of the rotor and the spin rate are kept constant. Therefore, the rate sensing equation is reduced to

(14) $$\begin{array}{l} \omega_{cx}=\frac{-T_{cy}}{h_{s}}+\frac{1}{h_{s}}\left[\frac{K_{x}+K_{y}}{2}\varphi_{y}-\left(I_{gt}-\frac{1}{2}I_{gs}\right)\omega_{s}^{2}\varphi_{y}-C_{g}\omega_{s}\varphi_{x}\right]\\ \omega_{cy}=\frac{T_{cx}}{h_{s}}-\frac{1}{h_{s}}\left[\frac{K_{x}+K_{y}}{2}\varphi_{x}-\left(I_{gt}-\frac{1}{2}I_{gs}\right)\omega_{s}^{2}\varphi_{x}+C_{g}\omega_{s}\varphi_{y}\right] \end{array}$$

### 3.2. Error Analysis of GyroWheel Rate Sensing

#### 3.2.1. Linearization Error

Since the rate sensing Equation (14) is obtained by linearizing the dynamic equation around the zero tilt operating point, it can be applied to measure spacecraft angular rates for small tilt conditions. Owing to the elimination of the higher order terms $F_{h.o.t}\left(x,\dot{x},\omega_{c},{\dot{\omega }}_{c}\right)$ in Equation (11), the rate sensing errors rise significantly when the GyroWheel is operated at large tilt angles.

The physical parameters of the GyroWheel system are presented in Table 1.

**Figure 3:**
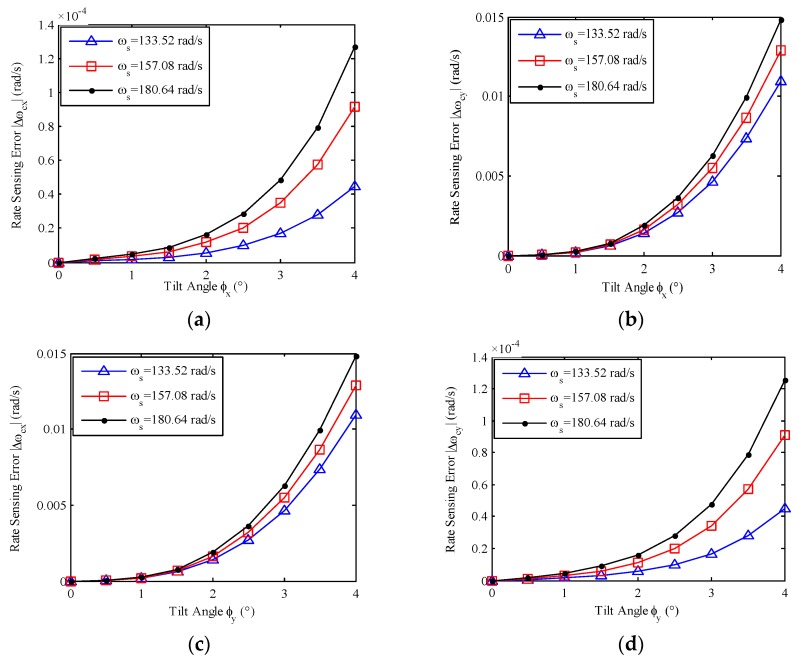
Relationship between rate sensing errors and tilt angles: (**a**) *X*-axis rate sensing error versus *x*-axis tilt; (**b**) *Y*-axis rate sensing error versus *y*-axis tilt; (**c**) *X*-axis rate sensing error versus *y*-axis tilt; (**d**) *Y*-axis rate sensing error versus *x*-axis tilt.

According to Table 1 and Equation (14), the relationship between the rate sensing errors and the tilt angles is shown in Figure 3.

According to Figure 3, we find:

1. The rate sensing errors caused by linearization at zero tilt are significantly correlated to the tilt angles of the GyroWheel rotor and the spin rate. The rate sensing errors increase with the increasing of the tilt angles and the increasing of the spin rate.
2. The rate sensing Equation (14) can be applied to measure spacecraft angular rates under small tilt conditions where the rotor tilt angles are less than 0.5°. However, when the GyroWheel is operated at a tilt angle of 4°, the rate sensing errors are up to 10^−2^ rad/s. Obviously, the rate sensing accuracy is far from satisfactory under large tilt conditions.
3. In an effort to ensure the rate sensing accuracy, the linearization errors should be compensated. The compensation terms are functions of tilt angles and spin rate, and can be denoted as $\delta_{nlx}\left(\varphi_{x},\varphi_{y},\omega_{s}\right),\delta_{nly}\left(\varphi_{x},\varphi_{y},\omega_{s}\right)$.

#### 3.2.2. Parameter Error

In Equation (14), the control torques $T_{cx},\;T_{cy}$ are proportional to the current in each torque coil. Generally, the control torque terms can be expressed in the following form:

(15) $$\frac{T_{cx}}{h_{s}}=k_{tx}i_{x},\;\frac{T_{cy}}{h_{s}}=k_{ty}i_{y}$$

where $i_{x},\;i_{y}$ are the currents in the torque coils, and $k_{tx},\;k_{ty}$ are torque factors. According to Equations (14) and (15), the estimate of spacecraft angular rate is dependent on the following types of terms:

1. Measurable values, including the tilt angles $\varphi_{x},\;\varphi_{y}$, the spin rate $\omega_{s}$, and the coil currents $i_{x},\;i_{y}$.
2. System parameters, including the moments of inertia $I_{gt},\;I_{gs},\;I_{rs}$, the stiffness coefficients $K_{x},\;K_{y}$, the damping coefficient $C_{g}$, and the torque factors $k_{tx},\;k_{ty}$.

In fact, the identification of the torque factors $k_{tx},\;k_{ty}$ is achievable by applying angular rate tests [27], therefore the control torque terms can be regarded as measurable values. However, the other system parameters are determined based on the material properties and the engineering CAD models used to machine the rotor and gimbal of the GyroWheel. Once the GyroWheel has been machined, there is not a more accurate method to identify these parameters. The parameter errors between their true values and design values will have a significant impact on the rate sensing accuracy. It is assumed that the true values of the system parameters have a deviation of ±10% from the design values given in Table 1. Substituting the true values and design values into Equation (14), the maximum rate sensing errors $\Delta \omega_{cxm},\;\Delta \omega_{cym}$ due to the individual parameter errors are listed in Table 2.

As seen in Table 2, the rate sensing errors due to the parameter errors are not negligible even under small tilt conditions. Based on the above analysis, the rate sensing method described by Equation (14) is not suitable for practical application.

## 4. GyroWheel Rate Sensing Using Genetic Algorithm Optimized Neural Networks

### 4.1. Rate Sensing Principle Based on Torque Balance Theory

As stated in Section 3.2.1, the compensation of linearization error is required to enhance the rate sensing accuracy of the GyroWheel system, especially under large tilt conditions. Incorporating the compensation terms into Equation (17), the rate sensing equation now becomes:

(16) $$\begin{array}{l} \omega_{cx}=\frac{-T_{cy}}{h_{s}}+\frac{1}{h_{s}}\left[\frac{K_{x}+K_{y}}{2}\varphi_{y}-\left(I_{gt}-\frac{1}{2}I_{gs}\right)\omega_{s}^{2}\varphi_{y}-C_{g}\omega_{s}\varphi_{x}\right]-\delta_{nlx}\left(\varphi_{x},\varphi_{y},\omega_{s}\right)\\ \omega_{cy}=\frac{T_{cx}}{h_{s}}-\frac{1}{h_{s}}\left[\frac{K_{x}+K_{y}}{2}\varphi_{x}-\left(I_{gt}-\frac{1}{2}I_{gs}\right)\omega_{s}^{2}\varphi_{x}+C_{g}\omega_{s}\varphi_{y}\right]-\delta_{nly}\left(\varphi_{x},\varphi_{y},\omega_{s}\right) \end{array}$$

Denote $T_{nly}=-h_{s}\cdot \delta_{nlx}\left(\varphi_{x},\varphi_{y},\omega_{s}\right),T_{nlx}=h_{s}\cdot \delta_{nly}\left(\varphi_{x},\varphi_{y},\omega_{s}\right)$, Equation (16) can be rewritten as follows:

(17) $$\begin{array}{l} \omega_{cx}=-\left\{\frac{T_{cy}}{h_{s}}-\frac{1}{h_{s}}\left[\frac{K_{x}+K_{y}}{2}\varphi_{y}-\left(I_{gt}-\frac{1}{2}I_{gs}\right)\omega_{s}^{2}\varphi_{y}-C_{g}\omega_{s}\varphi_{x}+T_{nly}\left(\varphi_{x},\varphi_{y},\omega_{s}\right)\right]\right\}\\ \omega_{cy}=\frac{T_{cx}}{h_{s}}-\frac{1}{h_{s}}\left[\frac{K_{x}+K_{y}}{2}\varphi_{x}-\left(I_{gt}-\frac{1}{2}I_{gs}\right)\omega_{s}^{2}\varphi_{x}+C_{g}\omega_{s}\varphi_{y}+T_{nlx}\left(\varphi_{x},\varphi_{y},\omega_{s}\right)\right] \end{array}$$

In Equation (17), the terms in the square brackets represent the spring torque, damping torque and coupling torque applied to the rotor of the GyroWheel, collectively called residual torques in this paper. More concretely, $\frac{K_{x}+K_{y}}{2}\varphi_{i}-\left(I_{gt}-\frac{1}{2}I_{gs}\right)\omega_{s}^{2}\varphi_{i},\;i=x,y$ represents the spring torque induced by the de-tuned stiffness, $C_{g}\omega_{s}\varphi_{i},\;i=x,y$ represents the damping torque, and $T_{nli},\;i=x,y$ includes part of the spring and damping torques ignored due to linearization, and the coupling torque induced by the non-zero tilts.

For a DTG, the tilt of the rotor with respect to its case is regulated about null, and it is operated in the tuned condition all the time. Therefore, the control torque required to maintain its zero tilt is a direct measure of the external angular rates of the DTG’s case about the two orthogonal axes lying in the rotor plane.

However, the GyroWheel is frequently operated in a de-tuned condition and at a non-zero tilt. The control torque required to hold the rotor spin axis at a constant tilt is a function of two independent effects: the torque induced by the external angular rates and the torque required to overcome the de-tuned stiffness, damping and coupling. Essentially, the rate sensing Equation (17) is an expression of torque balance. That is, the control torque applied to the rotor is equal to the sum of the precession torque due to the external rates and the residual torque due to the de-tuned spin rate and non-zero tilts.

As discussed in Section 3.2.2, the spring torque, the damping torque and the coupling torque vary with spin rate and tilt angles, and they cannot be measured directly. In addition, the torque factors are functions of tilt angles and spin rate [8]. Given that there is no feasible way to separate these torques in practical engineering, Equation (17) is rewritten as:

(18) $$\begin{array}{l} \omega_{cx}=-k_{ty}\left(\varphi_{x},\varphi_{y},\omega_{s}\right)i_{y}-\delta_{rex}\left(\varphi_{x},\varphi_{y},\omega_{s}\right)\\ \omega_{cy}=k_{tx}\left(\varphi_{x},\varphi_{y},\omega_{s}\right)i_{x}-\delta_{rey}\left(\varphi_{x},\varphi_{y},\omega_{s}\right) \end{array}$$

where $\delta_{rex},\;\delta_{rey}$ are equivalent rates due to the residual torques:

$$\begin{array}{l} \delta_{rex}\left(\varphi_{x},\varphi_{y},\omega_{s}\right)=-\frac{1}{h_{s}}\left[\frac{K_{x}+K_{y}}{2}\varphi_{y}-\left(I_{gt}-\frac{1}{2}I_{gs}\right)\omega_{s}^{2}\varphi_{y}-C_{g}\omega_{s}\varphi_{x}+T_{nly}\left(\varphi_{x},\varphi_{y},\omega_{s}\right)\right]\\ \delta_{rey}\left(\varphi_{x},\varphi_{y},\omega_{s}\right)=\frac{1}{h_{s}}\left[\frac{K_{x}+K_{y}}{2}\varphi_{x}-\left(I_{gt}-\frac{1}{2}I_{gs}\right)\omega_{s}^{2}\varphi_{x}+C_{g}\omega_{s}\varphi_{y}+T_{nlx}\left(\varphi_{x},\varphi_{y},\omega_{s}\right)\right] \end{array}$$

The method for external rate sensing given in Equation (18) depends on the good estimates of the equivalent rates $\delta_{rex},\;\delta_{rey}$ and the torque factors $k_{tx},\;k_{ty}$.

### 4.2. Identification of Torque Factors and Equivalent Rates

**Figure 4:**
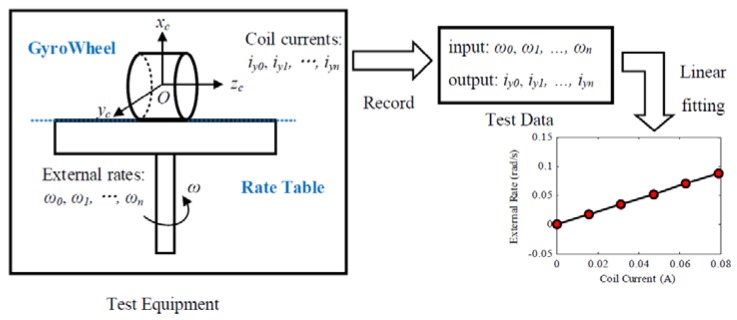
Schematic of angular rate test.

Angular rate test is a conventional method of torque factor identification for a DTG [27]. Given that the torque factor of the GyroWheel varies with spin rate and tilt angles, a series of angular rate tests are required. The main principle of the angular rate test is shown in Figure 4.

The GyroWheel is mounted on a single-axis rate table with its *x*-axis parallel to the rotation axis of the rate table. The GyroWheel is operated at a certain tilt angle $\left(\varphi_{xi},\;\varphi_{yi}\right)$ and spin rate $\omega_{si}$, and a series of external rates $\omega_{0},\;\omega_{1},\;\cdots ,\;\omega_{n}$ are provide by rotating the test table. Record the currents $i_{y0},\;i_{y1},\;\cdots ,\;i_{yn}$ in the *y*-axis torque coil.

Since the equivalent rate $\delta_{rex}$ is induced by the residual torque, it varies with spin rate and tilt angles rather than external rates. A set of equations can be obtained by substituting the test data into Equation (18):

(19) $$\left\{ \begin{array}{l} \omega_{0}=-k_{ty}i_{y0}-\delta_{rex}\\ \omega_{1}=-k_{ty}i_{y1}-\delta_{rex}\\ \begin{array}{cc} & \vdots \end{array}\\ \omega_{n}=-k_{ty}i_{yn}-\delta_{rex} \end{array}\right.$$

The test data can be processed by linear fitting. In this operating condition of the GyroWheel, the torque factor $k_{tyi}\left(\varphi_{xi},\varphi_{yi},\omega_{si}\right)$ and the equivalent rate $\delta_{rexi}\left(\varphi_{xi},\varphi_{yi},\omega_{si}\right)$ are determined by:

(20) $$\begin{array}{l} k_{tyi}\left(\varphi_{xi},\varphi_{yi},\omega_{si}\right)=\frac{\sum_{k=0}^{n}\left(-i_{yk}+{\bar{i}}_{y}\right)\left(\omega_{k}-\bar{\omega }\right)}{\sum_{k=0}^{n}{\left(-i_{yk}+{\bar{i}}_{y}\right)}^{2}}\\ \delta_{rexi}\left(\varphi_{xi},\varphi_{yi},\omega_{si}\right)=-\bar{\omega }-k_{tyi}\left(\varphi_{xi},\varphi_{yi},\omega_{si}\right){\bar{i}}_{y} \end{array}$$

Repeat the procedure with other operating conditions. Thus, a series of torque factor $k_{tyi}\left(\varphi_{xi},\varphi_{yi},\omega_{si}\right),i=1,2,\cdots ,p$ and equivalent rate $\delta_{rexi}\left(\varphi_{xi},\varphi_{yi},\omega_{si}\right),\;i=1,2,\cdots ,p$ can be obtained, where *p* represents the number of different operating conditions.

Similarly, the torque factor $k_{txi}\left(\varphi_{xi},\varphi_{yi},\omega_{si}\right),\;i=1,2,\cdots ,p$ and the equivalent rate $\delta_{reyi}\left(\varphi_{xi},\varphi_{yi},\omega_{si}\right),\;i=1,2,\cdots ,p$ can be identified by performing the angular rate test with the GyroWheel’s *y*-axis parallel to the rotation axis of the rate table.

### 4.3. Rate Sensing Using Genetic Algorithm Optimized Neural Networks

#### 4.3.1. Methodology: Genetic Algorithm Optimized Neural Network

The artificial neural network (ANN), a well-known soft computing tool, has wide-ranging applications and is often used to model the non-linear relationship between input parameters and output value(s). ANN technique is based on experimental data, so it can be applied to solve modelling problems where conventional approaches, such as regression analysis, fail or perform poorly. Multi-layer perception (MLP) ANN is widely utilized and is often trained with a back-propagation (BP) algorithm [28]. The BP algorithm is based on a learning rule by which the weights are evaluated in order to minimize the error between the neural network output and the desired output [21]. In addition, a global optimization algorithm, such as genetic algorithm (GA), should be added to the training process of the ANN algorithm to avoid local minima, which leads to false convergence of the ANN model.

As stated in Section 4.2, torque factors and equivalent rates are functions of tilt angles and spin rate. To realize rate sensing with the GyroWheel, it is essential to model the relationship between $\left(\varphi_{x},\varphi_{y},\omega_{s}\right)$ and $k_{tx},\;k_{ty},\;\delta_{rex},\;\delta_{rey}$. Since the model is significantly non-linear and complex, it is difficult to build the relationship based on theoretical derivations. Hence, the GA optimized ANN method is applied to the modelling problem in this study.

**Figure 5:**
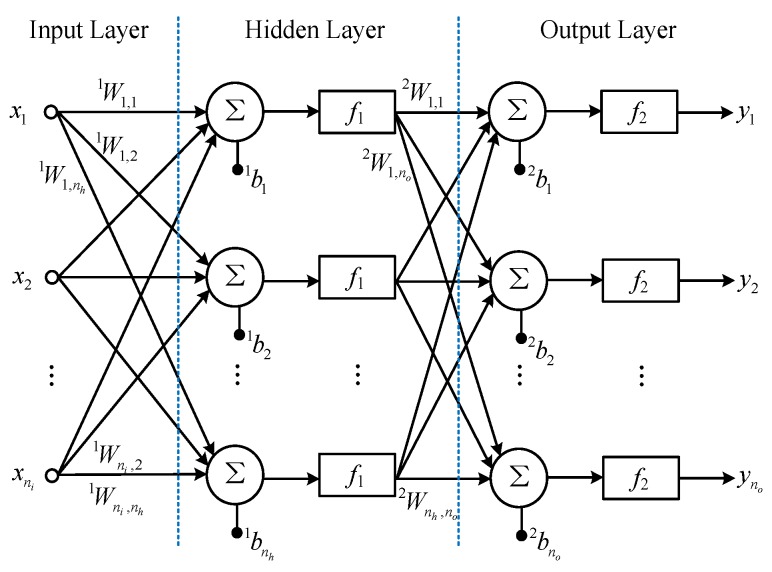
A simple MLP ANN.

According to the universal approximation theorem, a network with one hidden layer is sufficient to uniformly approximate any continuous and non-linear function [29]. A schematic of a simple MLP ANN is shown in Figure 5.

where $n_{i},\;n_{h},\;n_{o}$ are the numbers of inputs, hidden neurons and outputs, respectively. $x_{i},\;y_{j}$ represent the inputs and outputs of the network. Wi,j1 is the weight between the *i*-th input and the *j*-th hidden neuron, Wi,j2 is the weight between the *i*-th hidden neuron and the *j*-th output, and bi1,bj2 are bias values. $f_{1},\;f_{2}$ represent the activation functions. The outputs of the network can be expressed in the following form:

(21) yi=f2(bi2+∑k=1nhWk,i2f1(bk1+∑j=1niWj,k1xj)),i=1,⋯,no

The objective of the ANN is to minimize mean square error (MSE) described as follows:

(22) $$MSE=\frac{1}{p\cdot n_{o}}\sum_{i=1}^{n_{o}}\sum_{j=1}^{p}{\left(t_{ij}-y_{ij}\right)}^{2}$$

where *p* is the number of samples, *y* and *t* are predicted outputs of the ANN and targets, respectively.

**Figure 6:**
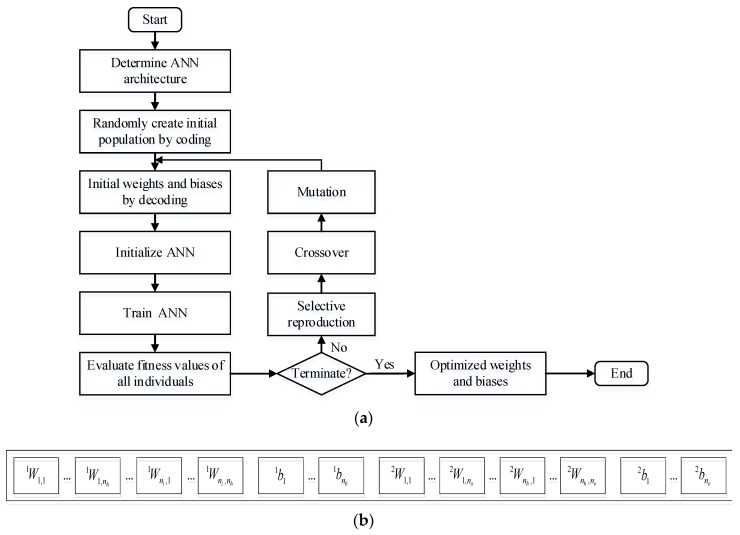
GA optimized ANN algorithm: (**a**) Flowchart; (**b**) An example of storing weights and biases of an ANN model in the genes of a chromosome.

In an attempt to improve the ANN’s performance, the initial weights and biases are optimized by GA. The GA optimized ANN algorithm is shown concisely in Figure 6.

As seen in Figure 6, the weights and biases of ANN model are stored in the genes of a chromosome. At the start of the algorithm, an initial population of individuals (also called chromosomes) is created.

The gene values are assigned to the initial weights and biases of the network, and the network is trained based on the BP algorithm. Then the fitness values of all the chromosomes of population are evaluated, the inverse of MSE is regarded as the fitness function. Later on, the more fit individuals are stochastically selected from the current population, and each individual’s genome is modified by crossover and mutation operations. These operations result in a new generation population of chromosomes. The generational process is repeated until any of the two termination conditions has been reached, i.e., the required number of generations has been reached, or convergence has been achieved. The weights and biases of the network are determined via a global optimization method, i.e., GA, which increases the performance of the ANN model.

#### 4.3.2. GAANN-Based Rate Sensing for GyroWheel

According to the analysis above, the rate sensing algorithm consists of the following steps:

Step 1. Experimental Data Collection and Data Preprocessing.

A database including 287 samples can be obtained by performing the test procedures as given in Section 4.2. The experimental data are recorded for 287 different operating conditions of the GyroWheel: 41 tilt conditions ($\varphi_{x}=\alpha \cos\lambda ,\;\varphi_{y}=\alpha \sin\lambda$, α=0°, 0.5°, 1°, 1.5°, 2°, 2.5°, 3°, 3.5°, 4°, λ=0°, 22.5°, 45°, 67.5°, 90°) and 7 different spin rates (133.52, 141.37, 149.23, 157.08, 164.93, 172.79, 180.64 rad/s). Of the 287 data sets, 229 records (80%) are randomly taken for the training process and the remaining 58 records are used for validation and testing (10% for each). The database has two parts: the input and the target. In this specific case, the inputs are: the tilt angles $\varphi_{x},\;\varphi_{y}$, and the spin rate $\omega_{s}$. To measure the external angular rates, the following target variables are used: the torque factors $k_{tx},\;k_{ty}$ and the equivalent rates $\delta_{rex},\;\delta_{rey}$.

In addition, the database must be normalized based on the activation functions used in the ANN architecture. In this study, all of the inputs and the targets are normalized between −1 and 1 by using the following equation:

(23) $$\begin{array}{l} {\bar{x}}_{ij}=\frac{2x_{ij}-\left(x_{i,max}+x_{i,min}\right)}{x_{i,max}-x_{i,min}},i=1,\cdots ,n_{i}{,}^{}j=1,\cdots ,p\\ {\bar{y}}_{ij}=\frac{2y_{ij}-\left(y_{i,max}+y_{i,min}\right)}{y_{i,max}-y_{i,min}},i=1,\cdots ,n_{o}{,}^{}j=1,\cdots ,p \end{array}$$

where $x_{ij},\;y_{ij}$ are the *i*-th input and output of the *y*-th sample respectively, the subscripts “*max*” and “*min*” represent the maximum and minimum values.

Step 2. Model Construction Using GA Optimized ANN.

Four independent neural networks are adopted to model the torque factors $k_{tx},\;k_{ty}$ and the equivalent rates $\delta_{rex},\;\delta_{rey}$ respectively.

In fact, the methods to find the best operating parameters for GA are mostly trial and error type [25]. Therefore, the parameters are selected after numerous experiments, and are listed in Table 3.

In addition, the choice of the number of the hidden neurons is of great importance to avoid over-fitting. An empirical formula is given below:

(24) $$n_{h}=\sqrt{n_{i}+n_{o}}+a$$

where a is an integer between 1 and 10. In addition, Belman-Flores recommended an over-fitting criterion for a network with one hidden layer [26], which defined an upper bound for the number of hidden neurons as:

(25) $$n_{h}\leq \frac{n_{t}}{4\left(n_{i}+1\right)}$$

where $n_{t}$ is the number of training sets. According to Equations (24) and (25), the number of hidden neurons is set as 10. The parameter settings of ANN is given in Table 4.

Based on the GA optimized ANN algorithm as shown in Figure 6, the four networks can be well trained, of which the weights and biases are adjusted so that each network may produce a desired output when a specific input is applied. The weights and biases are frozen and recorded after training, and the trained ANN models can be expressed in explicit forms:

(26) δrex=purelin(b112+∑j=110Wj,112tansig(bj11+∑i=13Wi,j11xi))δrey=purelin(b122+∑j=110Wj,122tansig(bj21+∑i=13Wi,j21xi))ktx=purelin(b132+∑j=110Wj,132tansig(bj31+∑i=13Wi,j31xi))kty=purelin(b142+∑j=110Wj,142tansig(bj41+∑i=13Wi,j41xi))

The pre-subscripts “1”, “2”, “3”, “4” in the weights and biases represent the ANN models of $\delta_{rex},\;\delta_{rey},\;k_{tx},\;k_{ty}$ respectively. The activation functions can be computed as:

(27) $$\begin{array}{l} purelin\left(x\right)=x\\ tansig\left(x\right)=\frac{2}{1+e^{-2x}}-1 \end{array}$$

Step 3. External Rate Sensing.

Combining Equations (18) and (26), the rate sensing equations can be expressed as follows:

(28) ωcx=−purelin(b142+∑j=110Wj,142tansig(bj41+∑i=13Wi,j41xi))⋅iy−purelin(b112+∑j=110Wj,112tansig(bj11+∑i=13Wi,j11xi))ωcy=purelin(b132+∑j=110Wj,132tansig(bj31+∑i=13Wi,j31xi))⋅ix−purelin(b122+∑j=110Wj,122tansig(bj21+∑i=13Wi,j21xi))

When the GyroWheel is used to measure spacecraft angular rates, the tilt angles of the rotor, the spin rate and the torque coil currents can be measured by the sensors. Substituting the values of tilt angles, spin rate and torque coil currents into Equation (28), the spacecraft angular rates can be determined.

#### 4.3.3. Simulation Results and Analysis

**Figure 7:**
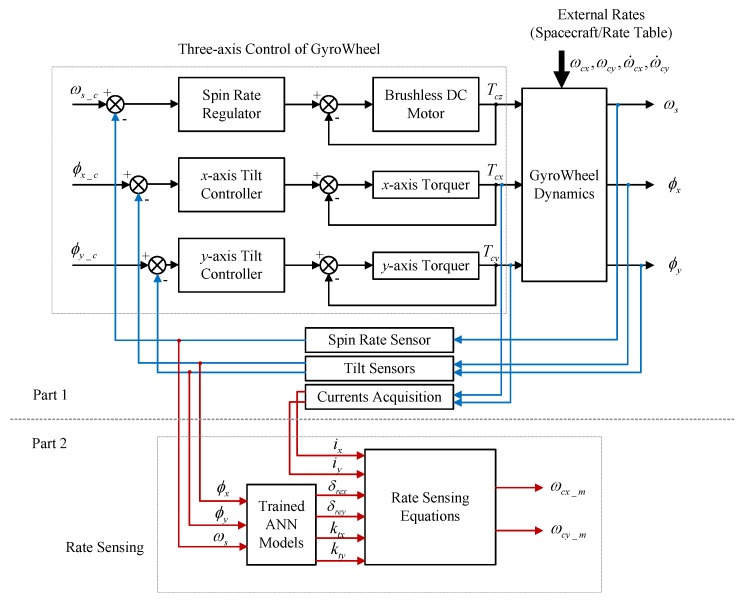
Schematic of the simulation platform.

In this section, the performance of the proposed rate sensing approach is investigated by numerical simulations. A schematic of the simulation platform is shown in Figure 7. $\omega_{s\_c},\;\varphi_{x\_c},\;\varphi_{y\_c}$ are the command inputs of the GyroWheel control loops, and $\omega_{s},\;\varphi_{x},\;\varphi_{y}$ are the corresponding measurable outputs of the three control loops. $\omega_{cx},\;\omega_{cy}$ represent the spacecraft angular rates or the external rates provided by the rate table, and $\omega_{cx\_m},\;\omega_{cy\_m}$ are the measured values of the external rates using the proposed rate sensing approach.

In Figure 7, Part 1 of the simulation platform is utilized to collect experimental data as stated in Section 4.3.2 (Step 1), which are used to train the four networks based on GA optimized ANN algorithm. Part 2 of the simulation platform is designed to realize the angular rate sensing of the spacecraft with the proposed method.

**Figure 8:**
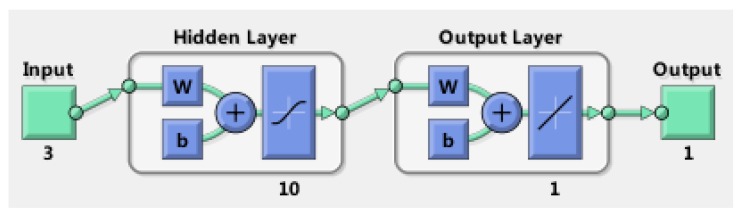
GAANN architecture for GyroWheel rate sensing.

**Figure 9:**
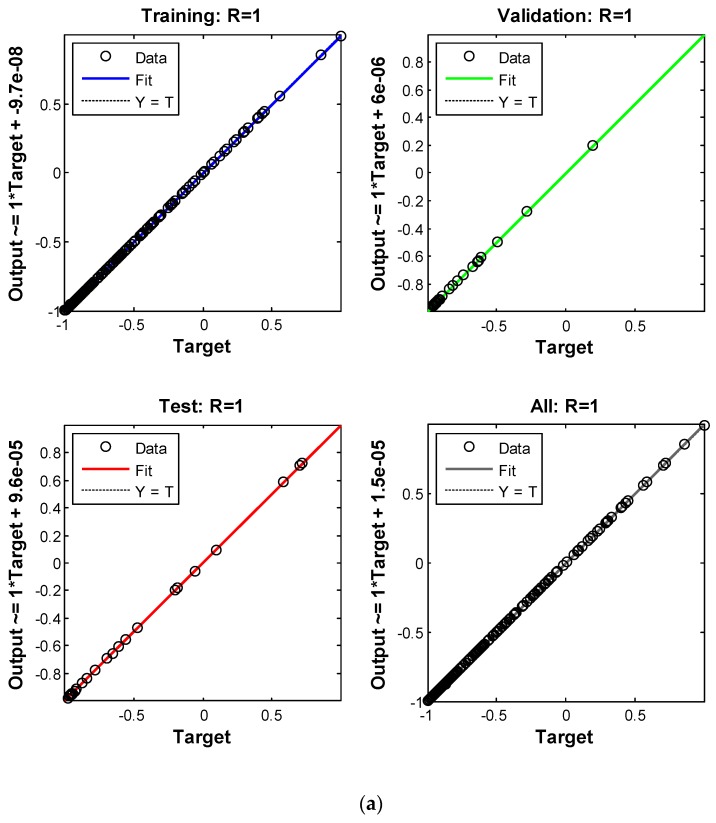

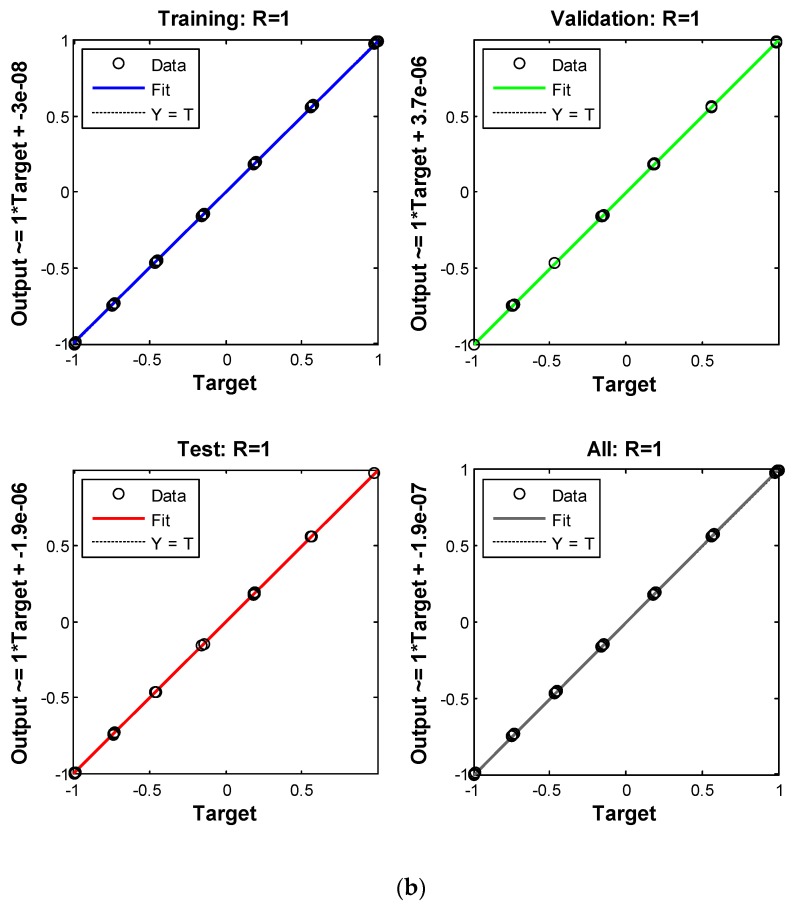
GAANN correlation performance: (**a**) ANN models for predicting equivalent rates; (**b**) ANN models for predicting torque factors.

The key parameters of the GyroWheel system are given in Table 1, and the parameters of the GAANN models are given in Table 3 and Table 4. The GAANN architecture is illustrated in Figure 8. After training, the performance of the ANN models are evaluated in terms of MSE and correlation coefficient, as shown in Table 5 and Figure 9. The MSE and correlation performances indicate that there is no over-fitting in the ANN models, and the ANN models can predict the torque factors and equivalent rates accurately.

The weights and biases of the final ANN models are presented in Table 6.

**Figure 10:**
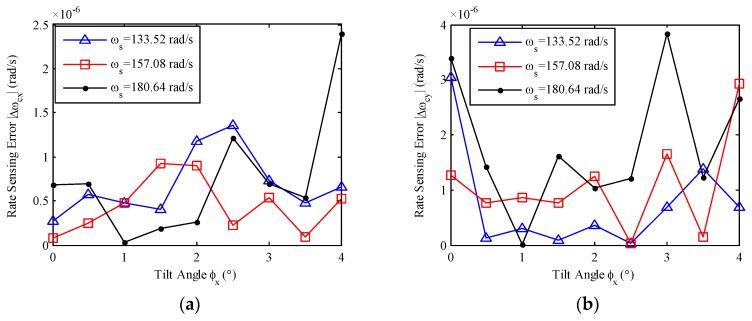

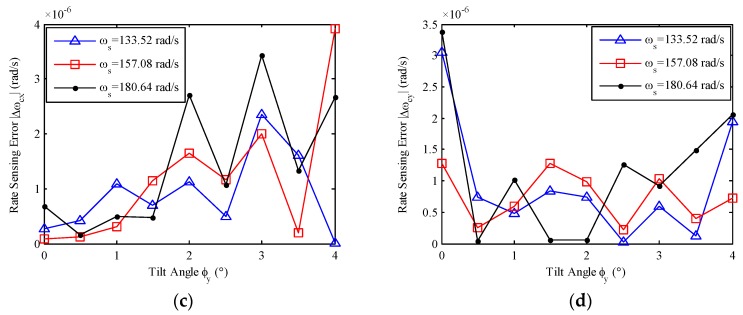
Relationship between rate sensing errors and tilt angles: (**a**) *X*-axis rate sensing error versus *x*-axis tilt; (**b**) *Y*-axis rate sensing error versus *y*-axis tilt; (**c**) *X*-axis rate sensing error versus *y*-axis tilt; (**d**) *Y*-axis rate sensing error versus *x*-axis tilt.

Under the same condition of Figure 3, the measured angular rates are calculated by substituting the weight and bias values into Equation (28), and the rate sensing errors are given in Figure 10.

In comparison with the traditional rate sensing method, the GAANN-based rate sensing method can effectively improve the measurement accuracy, even if the GyroWheel system is operated at a large tilt angle.

**Figure 11:**
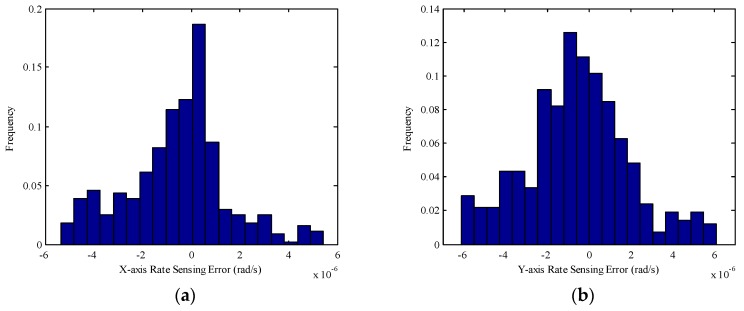
Histograms of Rate sensing errors: (**a**) *X*-axis; (**b**) *Y*-axis.

Furthermore, the rate sensing accuracy with the proposed method is verified under various operating conditions. 500 operating conditions are randomly determined, and the respective rate sensing errors are calculated, which are visualized in Figure 11 as histograms.

As seen in Figure 11, the rate sensing accuracy can reach 10^−6^ rad/s using the proposed method. Therefore, the rate sensing method given in Equation (28) is an effective way to estimate the spacecraft angular rates under various operating conditions.

In addition, by using more experimental data to train the ANNs, the accuracy of the GAANN-based rate sensing method can be further improved.

## 5. Conclusions

In this paper, the angular rate sensing problem is investigated for GyroWheel, a novel attitude determination and control device. A practical rate sensing method is developed by using the genetic algorithm optimized artificial neural networks. Compared with the traditional rate sensing method, the salient features of the proposed method are as follows:

1. The GAANN-based method provides a high rate sensing accuracy even under large tilt conditions. Therefore, it can be applied to measure angular rates in the whole operating range of the GyroWheel.
2. The GAANN-based method does not depend on the GyroWheel parameters that are difficult to identify. Instead, explicit ANN models are established using experimental data. Once the weights and biases of the ANN models are determined, the spacecraft angular rates can be estimated with the measurable tilt angles, spin rate and coil currents of the GyroWheel.

Limited to the experimental conditions, the effectiveness of the proposed method is verified by simulations rather than experiments with the GyroWheel prototype. In future research, the performance of the proposed method will be investigated for the GyroWheel prototype.

## Figures and Tables

**Table 1:** Physical parameters of GyroWheel system.

Parameters	Values
Rotor transverse inertia *I_rt_*	3.458 × 10^−3^ kg·m^2^
Rotor spin inertia *I_rs_*	6.402 × 10^−3^ kg·m^2^
Gimbal transverse inertia *I_gt_*	1.276 × 10^−5^ kg·m^2^
Gimbal spin inertia *I_gs_*	1.805 × 10^−5^ kg·m^2^
Stiffness coefficients *K_x_*, *K_y_*	0.092 Nm/rad
Damping coefficient *C_g_*	3.100 × 10^−8^ Nm/(rad/s)
Tile range $\varphi =\sqrt{\varphi_{x}^{2}+\varphi_{y}^{2}}$	0°≤φ≤4°
Range of spin rate $\omega_{s}$	133.52 rad/s ≤ $\omega_{s}$ ≤ 180.64 rad/s

**Table 2:** Rate sensing errors due to parameter errors.

Parameters	Small Tilt (φ=0.5°)		Large Tilt (φ=4°)	
	$\left|\Delta \omega_{cxm}\right|\left(\mathrm{rad}/s\right)$	$\left|\Delta \omega_{cym}\right|\left(\mathrm{rad}/s\right)$	$\left|\Delta \omega_{cxm}\right|\left(\mathrm{rad}/s\right)$	$\left|\Delta \omega_{cym}\right|\left(\mathrm{rad}/s\right)$
*I_gt_*	3.137 × 10^−4^	3.137 × 10^−4^	2.510 × 10^−3^	2.510 × 10^−3^
*I_gs_*	2.220 × 10^−4^	2.220 × 10^−4^	1.776 × 10^−3^	1.776 × 10^−3^
*I_rs_*	1.019 × 10^−4^	1.019 × 10^−4^	8.150 × 10^−4^	8.150 × 10^−4^
*K_x_*	4.690 × 10^−5^	4.690 × 10^−5^	3.752 × 10^−4^	3.752 × 10^−4^
*K_y_*	4.690 × 10^−5^	4.690 × 10^−5^	3.752 × 10^−4^	3.752 × 10^−4^
*C_g_*	4.220 × 10^−9^	4.220 × 10^−9^	3.376 × 10^−8^	3.376 × 10^−8^

**Table 3:** Parameter settings of GA.

Parameters	Values
Coding type	Real coding
Population size	100
Iterations	50
Selection operator	Roulette-wheel selection
Crossover probability	60%
Mutation probability	0.5%

**Table 4:** Parameter settings of ANN.

Parameters	Values
Number of hidden neurons	10
Epochs	2000
Training algorithm	Bayesian regulation back-propagation
Activation function of hidden layer	tan-sigmoid
Activation function of output layer	purelin (linear transfer function)

**Table 5:** MSE performance of GAANN models.

ANN	MSE Values		
	Training	Validation	Testing
1	1.1142 × 10^−8^	7.3956 × 10^−9^	1.5940 × 10^−8^
2	7.7689 × 10^−9^	1.6244 × 10^−8^	1.0707 × 10^−8^
3	1.7201 × 10^−9^	9.4487 × 10^−10^	7.4277 × 10^−10^
4	5.8538 × 10^−10^	8.4108 × 10^−10^	1.2611 × 10^−9^

**Table 6:** Weights and biases of ANN models.

ANN	Weights between Input and Hidden Layer	Biases of Hidden Layer	Weights between Hidden and Output Layer	Biases of Output Layer
1	0.0100, 0.5545, 0.0777; 0.1455, 0.4150, −0.1270; 0.0704, −0.3346, −0.0711; −0.0391, −0.7435, −0.0994; 0.1792, −0.4262, 0.0458; 0.2559, −0.0055, −0.0170; 0.2264, −0.2593, 0.0561; −0.2273, −0.3106, 0.0165; 0.2551, 0.2819, 0.0432; −0.2071, 0.2051, 0.0241.	−0.5267; −1.4775; −0.3092; 1.7672; −1.4598; −0.7754; 0.4807; −0.6000; −0.2493; −0.1981.	5.0975, −7.1387, −8.8096, −9.8384, −7.3076, 10.6156, −5.8899, 6.0972, −5.9602, −16.2299.	3.7141
2	0.5141, −0.3526, 0.0901; 0.2502, 0.0662, −0.0692; −0.7629, 0.4477, 0.0198; 0.3041, 0.1787, −0.0073; −0.6078, −0.2979, −0.1073; 0.3246, 0.1595, −0.0853; −0.4315, 0.3101, −0.0164; 0.1087, −0.0002, 0.1244; −0.4581, 0.2426, 0.0308; 0.2989, −0.0997, −0.2243.	−1.9411; 0.8813; −1.1333; 0.3888; 1.4684; −0.1584; −0.8051; −0.1484; −0.2198; −1.6234.	−5.3511, −10.8303, −1.5636, 8.1233, 4.6847, −4.7499, 4.5676, −4.1065, −3.9801, 2.6404.	−2.1126
3	0.1551, 0.0128, 0.2639; 0.0151, 0.0003, −0.3749; −0.3603, −0.0390, −0.4786; 0.0009, 0.0318, −0.3257; 0.0303, 0.0207, −0.9521; 0.0565, 0.0221, 0.2269; −0.1718, −0.0120, −0.2306; −0.0226, −0.0192, 0.6862; 0.0200, −0.1314, −0.2398; −0.0373, −0.0052, 0.2146.	0.1712; 0.2737; 0.1333; 0.3065; −1.7030; −0.2572; −0.5986; 0.4407; −1.0285; −0.1963.	0.5813, 0.6213, 0.0341, 0.5309, 0.9480, −0.4722, 0.6712, −0.4977, 0.2949, −0.4503.	0.8952
4	0.0064, −0.0065, 0.3470; 0.0150, −0.0038, 0.6597; 0.0137, −0.0044, 0.8656; −0.0123, −0.0312, 0.1601; −0.0019, 0.0316, −0.0801; 0.0036, −0.0433, −0.3119; −0.0196, 0.1975, 0.0048; −0.1069, −0.0316, 0.1904; −0.0218, 0.1599, 0.1726; 0.0258, −0.0250, 0.2503.	−0.1904; 0.4451; 1.6821; −0.3732; 0.1029; 0.1148; 0.4758; 0.9927; 0.0577; −0.2785.	−0.5990, −0.4535, −1.1659, −0.6272, 0.2505, 0.5259, −0.3702, −0.4228, 0.2553, −0.5580.	1.0207

## Abbreviations

The following abbreviations are used in this manuscript:

DTG	Dynamically Tuned Gyroscope
ANN	Artificial Neural Network
GA	Genetic Algorithm
CMG	Control Moment Gyroscope
DC	Direct Current
MLP	Multi-layer Perception
BP	Back-propagation
GAANN	Genetic Algorithm Optimized Neural Network
